# Systematics and relationships of *Tryssophyton* (Melastomataceae), with a second species from the Pakaraima Mountains of Guyana

**DOI:** 10.3897/phytokeys.136.38558

**Published:** 2019-12-10

**Authors:** Kenneth J. Wurdack, Fabián A. Michelangeli

**Affiliations:** 1 Department of Botany, MRC-166 National Museum of Natural History, Smithsonian Institution, P.O. Box 37012, Washington, DC 20013-7012, USA National Museum of Natural History, Smithsonian Institution Washington United States of America; 2 The New York Botanical Garden, 2900 Southern Blvd., Bronx, NY 10458, USA The New York Botanical Garden New York United States of America

**Keywords:** Guyana, leaf anatomy, Melastomataceae, molecular phylogeny, seeds, Sonerileae

## Abstract

The systematics of *Tryssophyton*, herbs endemic to the Pakaraima Mountains of western Guyana, is reviewed and *Tryssophytonquadrifolius* K.Wurdack & Michelang., **sp. nov.** from the summit of Kamakusa Mountain is described as the second species in the genus. The new species is distinguished from its closest relative, *Tryssophytonmerumense*, by striking vegetative differences, including number of leaves per stem and leaf architecture. A phylogenetic analysis of sequence data from three plastid loci and Melastomataceae-wide taxon sampling is presented. The two species of *Tryssophyton* are recovered as monophyletic and associated with mostly Old World tribe Sonerileae. Fruit, seed and leaf morphology are described for the first time, biogeography is discussed and both species are illustrated.

## Introduction

The flora of Guyana contains about 290 taxa of Melastomataceae in 40 genera, including three small endemic genera, *Maguireanthus* Wurdack, *Ochthephilus* Wurdack and *Tryssophyton* Wurdack (Wurdack 1993, Funk et al. 2007). These endemics are poorly known herbs from wet slope-forests in the Pakaraima Mountains, a region that is rich in Guiana Shield biota. *Boyania* Wurdack had been an additional Pakaraima Mountains endemic genus, based on *B.ayangannae* Wurdack, until a second species (*B.colombiana* Humberto Mend.) was unexpectedly discovered in Colombia (Mendoza-Cifuentes 2010). *Tryssophyton* was described in 1964 by John Wurdack as a very distinct monotypic genus that he assigned to tribe Bertolonieae. As a small, rhizomatous herb with a single stem crowned by a cluster of 10–16 narrow leaves, it vegetatively presents an atypical melastome, even amongst the highly variable, mostly herbaceous Bertolonieae. However, Bertolonieae, as traditionally circumscribed (i.e. sensu lato), has been shown to be polyphyletic with genera formerly assigned to it resolved within the Merianieae and two other distinct clades (Michelangeli et al. 2011, Goldenberg et al. 2015). The tribe has been recently narrowly circumscribed to contain only *Bertolonia* Raddi (Bacci et al. 2019).

*Tryssophyton* has been little studied since its description and collections have remained few. A 2012 expedition in the Pakaraima Mountains to reach the botanically unexplored ca. 1700 m summit of Kamakusa Mountain yielded new collections of the type species, *T.merumense* Wurdack, at lower elevations and a novel new species at the summit, which is described herein. We also provide further observations on the morphology and relationships of both species. Kamakusa Mountain and its vicinity at the wet, eastern edge of the Pakaraima Mountains have yielded many new plant taxa from the two expeditions (1960, 2012) that have traversed this remote region and it deserves further scientific exploration (Wurdack et al. 2013, Wurdack 2017).

## Materials and methods

In order to ascertain the phylogenetic position of both species of *Tryssophyton*, we sequenced plastid *rbcL* and/or *ndhF* following protocols and primers used in previous broad studies of the Melastomataceae (i.e. Clausing and Renner 2001, Michelangeli et al. 2011, 2014, Goldenberg et al. 2012, 2015). These data were analysed in the context of a family-wide taxon sampling for three plastid loci, *ndhF*, *rbcL* and *rpl16* (last locus treated as missing data for *Tryssophyton*) that included six representatives of other families of Myrtales as outgroups (see Appendix 1). All other sequences were obtained from GenBank (https://www.ncbi.nlm.nih.gov/genbank/) and mostly published in previous studies (Clausing and Renner 2001, Michelangeli et al. 2011, 2014, Goldenberg et al. 2012, 2015, Zeng et al. 2016), although a few have not been previously cited. Contigs were assembled in Geneious ver. 11 (http://www.geneious.com, Kearse et al. 2012) and alignments were performed with MUSCLE (Edgar 2004) as implemented through the Geneious plugin with the following parameters: maximum trees to build 20, optimal and diagonal optimisations on, anchor spacing 32 and minimum length of 24. The loci were analysed separately for quality control and then as a concatenated 3-locus analysis with no character exclusion sets. A maximum likelihood (ML) analysis was performed with RAxML ver. 8 (Stamatakis 2014) as implemented on CIPRES XSEDE (https://www.phylo.org/) with each locus as a separate partition. Clade support was estimated by bootstrap percentages (BP) using the same search conditions on 1000 ML replicates.

Scanning electron microscopy (SEM) used a Zeiss EVO MA15 (Carl Zeiss SMT, Inc., Peabody, Massachusetts) at 6–12 kV after sputter-coating herbarium specimen seeds or critical point dried (CPD) field-fixed (in ethanol) leaves with 25 nm of C + Au/Pd using a Leica EM ACE600 (Leica Microsystems GmbH, Wetzlar, Germany). Leaves were examined for venation after clearing in ethanol or 2.5% sodium hydroxide. For internal structure, they were hand cut for SEM or, for light microscopy (LM), they were paraffin-embedded, sectioned at 10 μm, stained with toluidine blue O and examined with a Zeiss Universal Compound Microscope. The anatomy of the delicate leaves was more intact with CPD and SEM than after traditional thin sectioning from the same starting material, thus our observations are largely based on SEM.

## Phylogenetic results

**Figure 1.**
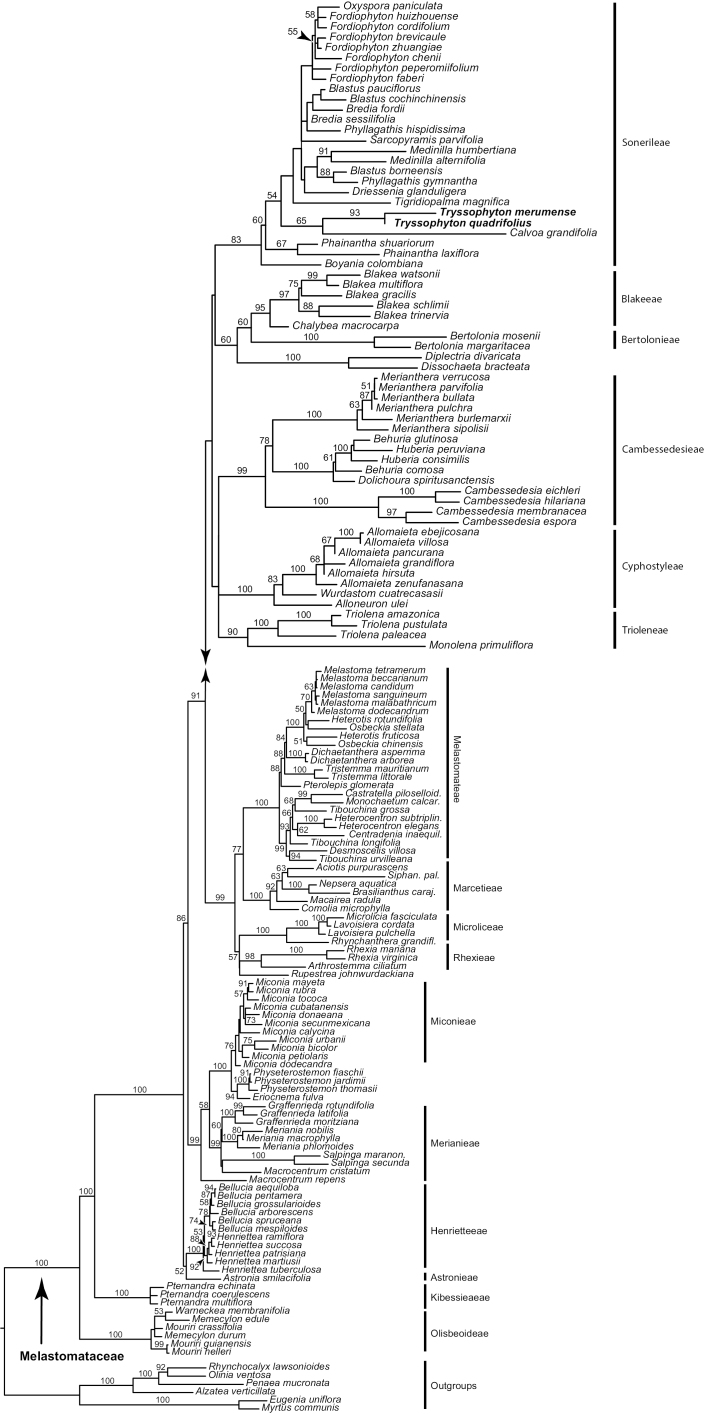
Relationships of *Tryssophyton* in a 3-locus phylogeny of Melastomataceae. Maximum likelihood tree with bootstrap support values based on analysis of plastid *ndhF*, *rbcL* and *rpl16* data.

Melastomataceae are resolved as a strongly supported (BP 100) family with mixed backbone support, which of relevance here was notably weak (BP 60 or less) amongst the deepest nodes of Sonerileae (Fig. 1). Both species of *Tryssophyton* form a strongly supported clade (BP 93) that is weakly resolved (BP 65) with African *Calvoa* near the base of the Sonerileae-Dissochaeteae complex (sensu Renner et al. 2001). *Boyania* is weakly supported (BP 60) as the earliest diverging member of Sonerileae. Marcetieae are sister to Melastomateae and distant from *Tryssophyton*.

## Taxonomic treatment

### Tryssophyton quadrifolius

Taxon classificationPlantaeMyrtalesMelastomataceae

K.Wurdack & Michelang. sp. nov.

7C7CEAB5-B2F6-5438-88A1-374D23F66126

urn:lsid:ipni.org:names:77203430-1

Figure 2

#### Diagnosis.

Differs from *Tryssophytonmerumense* in 4-verticillate, ovate, petiolate leaves, versus 10–16 verticillate, oblanceolate-lanceolate, subsessile leaves.

#### Type.

Guyana. Cuyuni-Mazaruni Region: Summit of Kamakusa Mtn. (i.e. on top of 4^th^ escarpment of four); impenetrable elfin forest to 3 m, extremely dense and wet, rich in epiphytes, with *Bonnetia* (2 spp.), Brocchiniacf.tatei, Malpighiaceae, Melastomataceae, Cyperaceae spp., *Weinmannia*, Ilexcf.retusa, 5°52'50.9"N, 60°6'11.7"W, 1691 m elev., 8 June 2012 (fl.), *K. Wurdack 5865* with E. Tripp, A. Radosavljevic, and J. Ralph (holotype: BRG!, isotype: US-3731242!).

#### Description.

***Habit*** perennial, rhizomatous herbs; rhizomes persistent, fleshy, horizontal, to 10 cm long, 3–6 mm diam., rarely branched, bearing 1–2 leafy, erect, aerial stems per axis and older cup-shaped (collar to 0.3 mm high and centre sunken) aerial stem scars; adventitious roots fine, fibrous. ***Indument*** sparse on aerial stems, leaves, pedicels, hypanthium and sepal margins; trichomes simple, to 0.1 mm long, glandular, reddish. ***Aerial stems*** 8.5–11.5 cm tall, 0.9–2 mm diam., terete, purple, slightly flared at rhizome attachment, summit with cluster of leaves and often 1 secondary branch 1–2 cm long and crowned by another cluster of leaves. ***Leaves*** 4-verticillate, opposite pairs sub-equal in size, simple, petiolate, exstipulate; petioles 2–5 mm long (of slightly subequal length within whorl), ca. 1 mm diam.; lamina 2.1–5.2 × 1.0–1.9 cm, length:width ratio 2.2–3.7:1 (mean = 3.0, SD = 0.50, *n* = 11), symmetrical, ovate to lanceolate, membranous, apex acuminate, base cuneate, margin minutely serrate, with 6–12 teeth per side (2–5 teeth/cm); teeth 0.5–0.9 × 0.1 mm, first-order, spacing regular, projecting 0.2–0.3 mm from margin, sinus shape rounded, distal flank concave and proximal flank straight, apex long-attenuate; leaf tip with 1–3(13) adaxial scales, 0.6–1 × 0.1 mm, similar to attenuate teeth apices. ***Venation*** suprabasal acrodromous, with one pair of major (costal) secondaries 1/2 of the gauge of the midvein, joining the midvein 0.5–1 mm above the leaf base; and one pair of intramarginal secondaries < 1/3 of the gauge of the midvein (poorly defined from tertiary thickness), joining < 0.5 mm from base, traversing 0.2–0.8 mm from margin, distally fading into exterior tertiary loops; up to 12 interior tertiary veins per side, quaternaries random reticulate. ***Inflorescence*** terminal, pedunculate, bearing 1–4 flowers; peduncle 2.2–2.6 cm long, 0.5–0.7 mm diam. mid-length, terete, flaring at base, purple; bracts persistent, lanceolate, 1–1.3 × 0.3–0.4 mm, apex apiculate, margin entire. ***Flowers*** 4-merous, bisexual, pedicellate; pedicels 8.5–11.5 mm long, 0.4–0.6 mm diam. mid-length, terete, purple; hypanthium at anthesis ca. 2.5 mm × 2–2.5 mm (excluding calyx), campanulate, obscurely costate with thin ribs; calyx lobes 4, in bud narrowly triangular, ascending, protective of young corolla, at anthesis spreading, 0.8–1 mm tall, broadly triangular above short calyx tube ca. 0.4 mm high; petals ca. 9.5 × 5 mm, margins entire, in vivo red outside and pink inside. ***Stamens*** 8, incurved in bud with tips extending into hypanthium below point of filament attachment, slightly anisomorphic, with the antesepalous whorl larger than the antepetalous, glabrous; filaments 5–6 (antesepalous) or 4 (antepetalous) mm long, 0.2 mm diam., linear, pink in vivo; thecae 4.5 or 3.5 mm long, basifixed, connective basally prolonged, dilated below thecae, forming a thickened annulus that is more or less ventrally bilobate, thickening 0.8 or 0.5–0.6 mm diam., yellow in vivo; anther 0.1–0.2 mm wide at terminal pore. ***Ovary*** superior, ca. 1.8 × 1.3–1.5 mm, glabrous. ***Style*** ca. 10 mm long, 0.2 mm diam., curved, glabrous; stigma punctiform, minutely papillose. ***Capsule*** ovate, ca. 3 × 3.5 mm, crowned by persistent calyx. ***Seeds*** ovoid, ca. 0.7 × 0.4 mm (immature and partly collapsed), sparsely papillose, brown.

#### Etymology.

The specific epithet is derived from *quadri*- (Latin, four) and *folium* (Latin, leaf) and refers to the 4-verticillate leaves.

#### Distribution and ecology.

*Tryssophytonquadrifolius* is only known from the summit of Kamakusa Mountain where it was encountered as an infrequent epiphyte on moss and lichen covered branches and trunks of large shrubs. Scattered small plants with single stems were observed sporadically along the main trail transect cut along the north-south orientated narrow summit. The few reproductive plants were in a more sheltered spot on the western edge of the summit on a branch trail that was cut to a cliff edge for a view. Flowers and young fruit were collected in June. The summit of Kamakusa Mountain is covered by low montane evergreen cloud forest, 3–4 m tall, with dense thickets dominated by *Bonnetiatepuiensis* Kobuski & Steyerm. and *B.roraimae* Oliv. (Bonnetiaceae), *Byrsonimapachypoda* W.R. Anderson (Malpighiaceae), *Miconiaacutifolia* Ule and *M.silicicola* Gleason (Melastomataceae), *Raveniopsismicrophyllus* K. Wurdack (Rutaceae) and species of *Weinmannia* L. (Cunoniaceae) on a peat substrate overlying sandstone. Vascular epiphytes included species of *Utricularia* L. (Lentibulariaceae), Bromeliaceae and Orchidaceae.

#### Conservation status.

While the upper part of Kamakusa Mountain is presently pristine and undisturbed, the new species is a delicate epiphytic herb with few reproductive plants (2 of 15 aerial stems collected had buds, flowers and/or fruit) in an area of extremely limited montane habitat. The species is vulnerable to climate and land use changes, such as regional gold mining (see Wurdack 2017). Following the criteria and categories of IUCN (2012, 2019) and similar to the recently described Kamakusa endemic *Raveniopsismicrophyllus*, *Tryssophytonquadrifolius* is given a preliminary status of Critically Endangered (CR) under geographic range criteria B1 (extent of occurrence < 100 km^2^ (B1) and area of occupancy < 10 km^2^ (B2a, number of locations =1; B2b, continuing decline projected).

**Figure 2.**
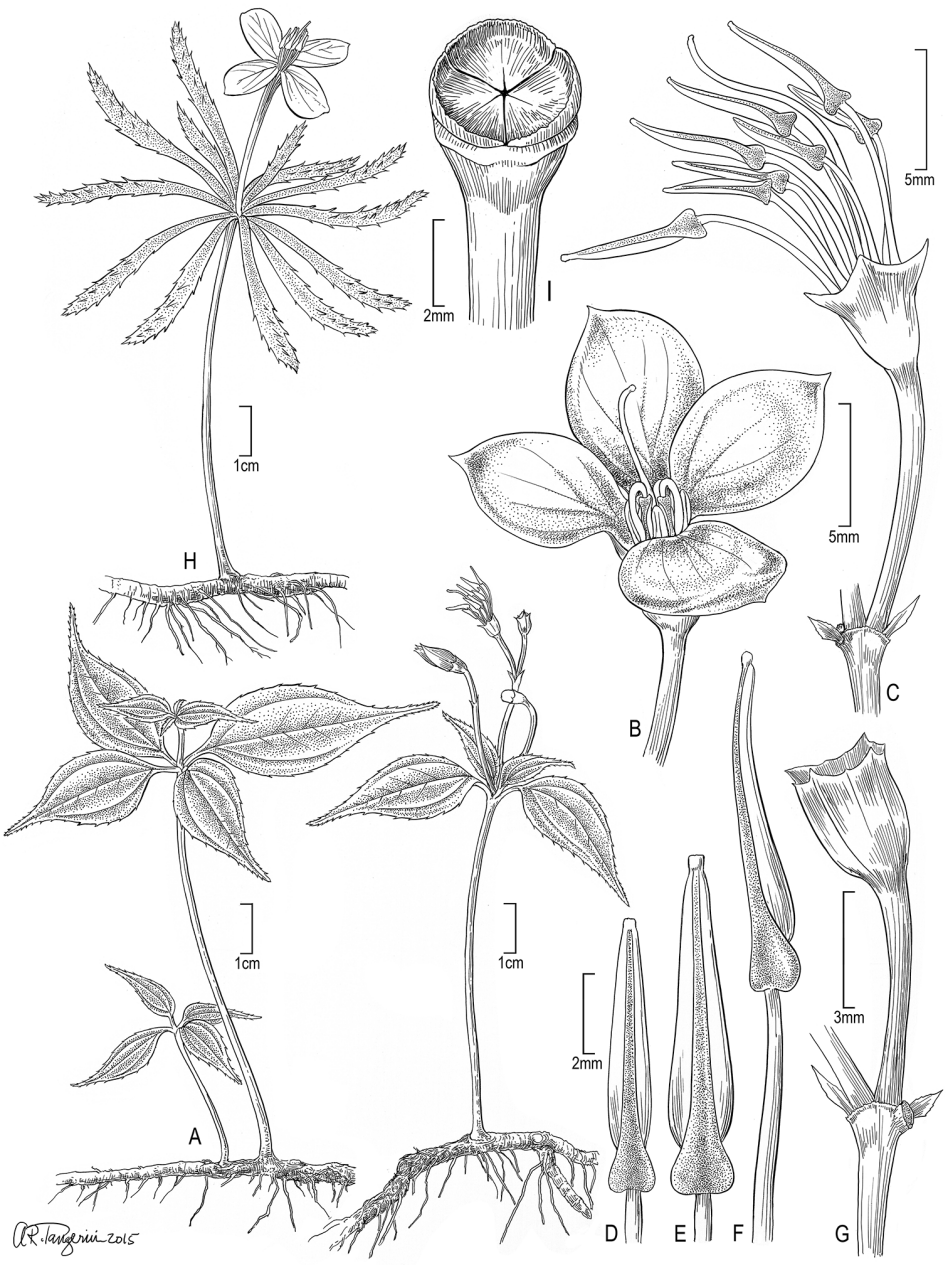
Illustration of *Tryssophytonquadrifolius* (**A–G**) and *T.merumense* (**H, I**). **A** Habit (right view in flower) **B** young flower with anthers inflexed **C** flower with anthers erect and petals fallen **D** shorter antepetalous anther, dorsal **E** longer antesepalous anther, dorsal **F** longer anther, lateral **G** young fruit **H** habit **I** capsular, 3-merous fruit. Sources: **A–G***Wurdack 5865***H***Radosavljevic 165***I***Wurdack 5870* (all US).

### Tryssophyton merumense

Taxon classificationPlantaeMyrtalesMelastomataceae

Wurdack, Mem. New York Bot. Gard. 10: 155. 1964.

DA842B5F-321A-5782-8FF2-13CA540661B9

#### Type.

Guyana. Cuyuni-Mazaruni Region, Partang River, Merume Mtns., Merume Mt.; common on mossy logs in forest, 1140 m elev., 4 Jul 1960 (fl., fr.), *S.S. Tillett*, *C.L. Tillett*, *& R. Boyan 43988* (holotype: US-2343844!; isotypes: K-000329332!, NY-00245868!).

It should be noted that both the US and NY sheets are each marked as the holotype. However, the protologue clearly states that the US specimen is the holotype, even citing the sheet number and has that designation in J. Wurdack’s handwriting. The NY sheet has “holotype” merely typed on the label. Thus, there is no need to lectotypify this name and the NY specimen should be considered as an isotype.

#### Etymology.

The genus is combined from *tryssos* (Greek, dainty or delicate) and *phyton* (Greek, plant) and refers to the plant habit. The specific epithet refers to Merume Mountain where the type was collected.

#### Additional collections examined.

Guyana. Cuyuni-Mazaruni Region: Pakaraima Mountains, upper Karowrieng River at Maipuri Falls; mixed bryophyte, pteridophyte, herb community; sandstone boulders, white sand, large cave behind falls, 5°41'N, 60°13'W, 575–600 m elev., 13 Oct 1992 (fl.), *B. Hoffmann 2939* (NY!, US!). 2^nd^ and 3^rd^ escarpments (of four) of Kamakusa Mt., upper west-facing slopes below summit, rich forest with *Licania*, Ebenaceae, tree ferns, Arecaceae, 5°52'55.2"N, 60°6'34.5"W, 1330 m elev., 8 June 2012 (fl., fr.), *K. Wurdack 5870* (US!). Potaro-Siparuni Region: Mt. Wokomung, easternmost pinnacle of massif, scrub forest on sandstone and peat, with *Guadua*, *Euterpe*, and *Sphagnum*, 5°5'34.4"N, 59°50'13.3"W, 1524 m elev., 13 Jul 2003 (fl.), *H.D. Clarke 10822* (NY!, US!). Mt. Ayanganna, east slope, plateau above second escarpment, growing on mossy tree trunks and roots, 5°22'28"N, 59°58'06"W, 1340 m elev., 16 Mar 2014 (fl.), *A. Radosavljevic 165* (US!).

#### Distribution and ecology.

The five collections of *T.merumense* span a 90 km section of the central Pakaraima Mountains, but further exploration is likely to expand its range into similar habitats. The species was only recently discovered (*Radosavljevic 165*) on the slopes of relatively well-explored Mount Ayanganna, the highest mountain (2041 m) wholly within Guyana. At mid-elevations on the western slopes of Kamakusa Mountain, it occurred (*Wurdack 5870*) as scattered, rarely-reproductive individuals on rotting logs and peaty-humus zones around the bases of trees.

The mountainous area north of the village of Imbaimadai and including Kamakusa Mountain has been variously mapped as the Merume Mountains. However, exactly what corresponds to the peak “Merume Mountain” within the region and indicated as the type locality of *T.merumense*, is unclear. Field notes (Bassett Maguire Field Collections, vol. 19, Archives of The New York Botanical Garden) reveal that during 11 Jun–16 Jul 1960, after leaving Imbaimadai, the collecting team, led by Stephen Tillett entered the Kamakusa Mountain area from along the Partang River. After reaching Partang Falls, they ascended into the uplands following existing trails, which were probably made by gold-miners or “pork-knockers.” It is likely that “Merume Mountain” of Tillett et al. is equivalent to Kamakusa Mountain, but details referring to lower elevations, southeast ridge, southeast side and cliffs do not indicate they reached summit where *T.quadrifolius* was collected.

## Discussion

The broader relationships which we recovered within Melastomataceae largely agree with those from prior studies using the underlying sequence data (i.e. Clausing and Renner 2001, Michelangeli et al. 2011, 2014, Goldenberg et al. 2012, 2015, Zeng et al. 2016, Bacci et al. 2019). *Tryssophyton* is clearly monophyletic and a member of the Sonerileae-Dissochateae complex, although a sister-relationship with *Calvoa* Hook. f. has low support and resolution within the tribe is also poor. A weaker (BP < 50) placement with *Calvoa* was recently reported for *T.merumense*, based on ITS data (Bacci et al. 2019). *Calvoa* is a tropical African genus containing about 19 species of herbs to small, woody shrubs (Jacques-Félix 1981, Figueiredo 2001). *Calvoa* has funnelform and costate hypanthia that develop into apically dehiscent capsules and are features common to most genera that have been assigned to the Bertolonieae + Sonerileae (Renner 1993, Bacci et al. 2019). However, the staminal morphology of *Calvoa* with short pedoconnectives and dorsal appendages (Jacques-Félix 1981) does not resemble that of *Tryssophyton*. *Sarcopyramis* Wall. (not sampled here; see Bacci et al. 2019), a southeast Asian genus of one morphologically variable (*S.napalensis* Wall., sensu lato; Hansen 1978) or multiple species of herbs was found by Bacci et al. (2019) to also belong to this clade and is similar to *Calvoa* in details of stamen morphology and long-papillose stigmas.

The more robust placement of *Tryssophyton* within Sonerileae has clearer implications for taxonomy and historical biogeography. When originally describing *Tryssophyton*, J. Wurdack (1964) placed it in Bertolonieae, presumably based on its herbaceous habit and fruit morphology, but he also compared it to Asian and African members of the Sonerileae as possible relatives. Bertolonieae has been shown as widely polyphyletic (Bacci et al. 2019). Wurdack (1964) also compared the stamens of *Tryssophyton* to those of *Marcetia* DC., due to their elongated shape and lack of pedoconnectives or appendages. *Marcetia* (not sampled here but clearly affiliated with other genera included in the Marcetieae; see Rocha et al. 2016, 2018) is not related to *Tryssophyton* and its seed morphology is markedly different in being cochleate and large-tuberculate (Rocha et al. 2018: fig. 7T).

The great majority of the species in the Sonerileae-Dissochaeteae complex are found in the Old World. The exceptions are *Tryssophyton* and the Neotropical genera *Boyania* and *Phainantha*, resolved as successive branches, with poor support, at the base of the clade. *Boyania* contains two trailing (stoloniferous) or climbing herbaceous species with disjunct distributions. One of them, *Boyaniaayangannae*, also grows in the same region as *Tryssophyton*, while the second species, *Boyaniacolombiana*, is restricted to the easternmost slopes of the Colombian Andes (Mendoza-Cifuentes 2010). *Boyania* was also initially placed in the Bertolonieae, based on habit and fruit and anther morphology, but J. Wurdack remarked that, given its 5-locular ovaries, it was intermediate between New World Bertolonieae and Old World Sonerileae. Recent phylogenetic evidence has suggested that *Boyania* may not be monophyletic (Bacci et al. 2019), although both species are morphologically similar. *Phainantha* contains five mostly climbing woody species (trailing herb in *P.steyermarkii* Wurdack), four of which are found in the Guiana Shield and one in southern Ecuador from the Cordillera del Condor which has phytogeographic ties with the Guiana Shield (Wurdack 1993, Berry et al. 2001, Ulloa Ulloa and Neill 2006). *Phainantha* was initially unplaced when described by Gleason (1948) due to lack of seeds and subsequently Renner (1993) placed it with Merianieae in her family-wide morphological analysis of the Melastomataceae. The character evolution and biogeography implications with the resolution of these three neotropical genera at or near the base of the Sonerileae-Dissochaeteae complex are important for understanding the evolution of the entire clade. For example, there are notable differences in merosity; *Phainantha*, *Sarcopyramis* and *Tryssophyton* are 4-merous and *Boyania* and *Calvoa* are 5-merous. The support for the early-diverging nodes of this clade is presently poor and the taxonomic and geographical sampling is too small for firm conclusions. However, if this topology holds with increased sampling of taxa and genetic loci, it would suggest that the mostly Asian Sonerileae-Dissochaeteae complex originated in South America and then dispersed to Africa and later to Asia where it diversified. A similar pattern has been found to the Melastomateae (Michelangeli et al. 2013, Veranso-Libalah et al. 2017). Recent phylogenetic studies of the Sonerileae-Dissochaeteae complex have already shown that most genera are not monophyletic and the group needs considerable further study (Zeng et al. 2016, Zhou et al. 2019a, 2019b).

## Morphology of *Tryssophyton*

**Figure 3.**
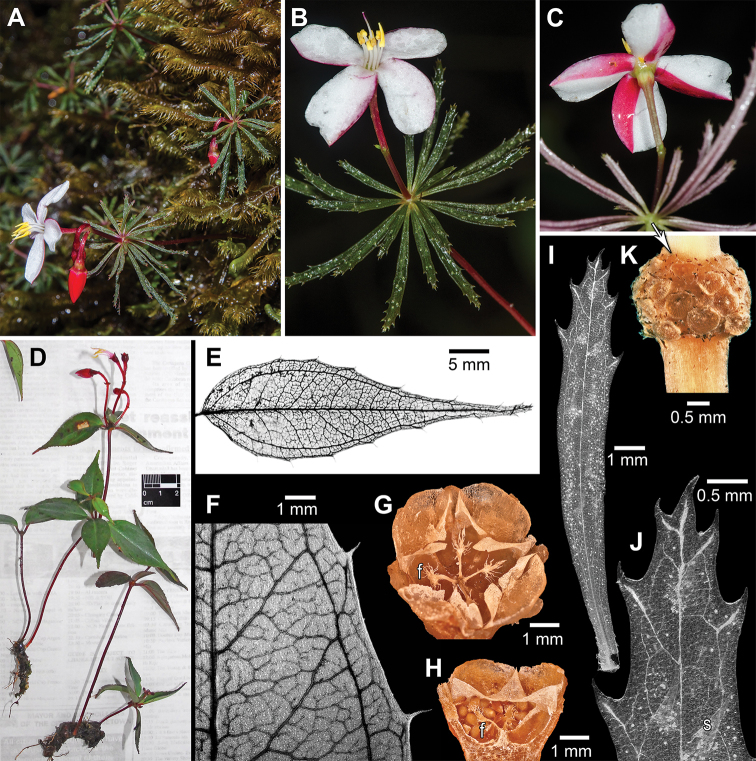
Macromorphology of *Tryssophytonmerumense* (**A–C, G–K**) and *T.quadrifolius* (**D–F**). **A** habit, growing with bryophytes and downward pointing red buds **B** flowering plant, adaxial **C** Flowering plant showing bicoloured petals, abaxial **D** part of type collection in vivo just before pressing **E** whole leaf venation, abaxial **F** venation close-up showing major veins and reticulate quaternaries, abaxial **G** Dehisced 4-merous fruit, with interior persistent fimbriate placenta remnants (f), axial (tips of 8 triangular valves partly broken) **H** fruit, with seeds, fimbriae (f) and carpel septa, transverse view **I** whole leaf venation and crystal druses (white spots), adaxial **J** close-up of leaf tip with marginal teeth and non-vascularised scales (s), adaxial **K** phyllotactic arrangement with leaf attachment scars surrounded by darkened glandular trichomes, lateral view with main stem at bottom, peduncle at top. Sources: **A–C, I–K***Radosavljevic 165***D–F***Wurdack 5865***G, H***Wurdack 5870* (all US).

Although reproductive features (flowers and fruit) and overall habit (rhizomatous herbs) are nearly identical between the two species, *Tryssophytonquadrifolius* is vegetatively easily distinguished by fewer leaves per stem and differences in leaf architecture (see Diagnosis; Figs 2, 3). Both species have short stems (6.5–11.5 cm tall), which are each crowned with a cluster of leaves; they sometimes possess additional leaf sets topping 1–5 shorter (1.5–3.5 cm tall) secondary branches arising from the first crown. This pattern of reiteration with secondary branches crowned by leaves has not been previously reported for *T.merumense*; it is not seen on the holotype but occurs on some plants in all other collections. In *T.quadrifolius*, each leaf cluster is comprised of two decussate pairs of opposite leaves that are nearly superposed so as to appear 4-verticillate. In *T.merumense*, the 10–16 leaves of varying sizes form a tight verticillate-like cluster and is likely a spiral decussate arrangement of multiple, nearly superposed leaf pairs. The rows (parastichies) of leaf attachment scars apparently form genetic spirals (Fig. 3K) and self-shading is largely absent (Fig. 3A, B). *Tryssophytonquadrifolius* resembles a typical melastome with regard to leaf form while *T.merumense* deviates with its narrow leaves, which have juvenile aspects of young leaves before full expansion. The leaves of *T.merumense* have been described as sessile (Wurdack 1964, 1993). The laminar base is finely tapering (attenuate) and decurrent, obscuring any defined petiole which is in marked contrast with *T.quadrifolius* where the petiole is well defined from the cuneate base. The venation of *T.quadrifolius* is suprabasal acrodromous with two sets of lateral major veins including a pair of costal secondaries diverging clearly above the leaf base and a pair of thinner intramarginal secondaries diverging closer to the base (Fig. 3E). It has well-developed higher order veins including tertiaries connecting the major veins and a ramified quaternary vein network and has simple teeth partly penetrated by a thickened principal vein (Fig. 3F). The venation of *T.merumense* is acrodromous with only a single pair of lateral major veins that traverse the attenuate leaf base (i.e. not suprabasal) through the length of the leaf and sparse higher order veins (Fig. 3H). The teeth (Fig. 3I, J) are similar, but shorter and the thickening of their principal vein is more pronounced than in *T.quadrifolius*. *Boyania*, *Calvoa* and *Sarcopyramis* usually have two pairs of lateral major veins (prominent costal and thinner intramarginal secondaries) that begin at the leaf base. *Phainantha* is more diverse with variation in costal secondary vein position relative to the margin and often well-developed intramarginal veins. In *Phainanthashuariorum* C. Ulloa & D.A. Neill (*Palacios et al. 8565*, US), the costal veins are so close to the margins so as to appear nearly marginal; however a narrow pair of intramarginal veins remains along the lower edges before distally merging with the adjacent costal veins.

**Figure 4.**
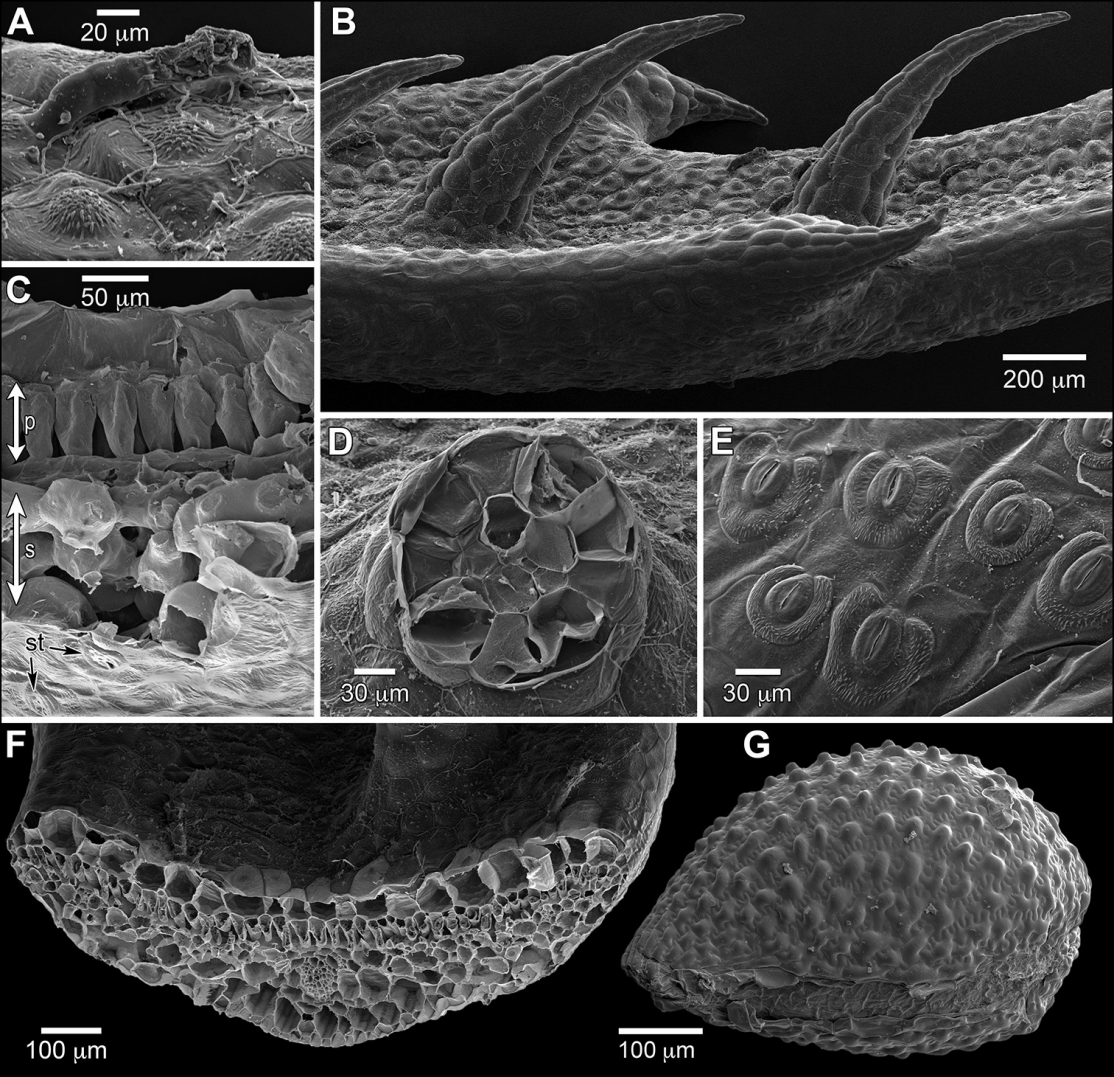
Micromorphology of *Tryssophytonmerumense*. **A** Leaf glandular trichome, adaxial **B** leaf with marginal teeth, erect adaxial scales and glandular trichomes, lateral view of distal part **C** leaf, with single file palisade parenchyma (p), loose spongy parenchyma (s) and stomata (st) beneath intercellular spaces, close-up of transverse view **D** leaf scale, transverse view near adaxial scale base **E** stomata, abaxial **F** leaf, transverse view **G** seed, lateral view with raphal zone near bottom. Sources: **A–F***Radosavljevic 165***G***Wurdack 5870* (all US).

While both *Tryssophyton* species grow on Kamakusa Mountain, they do not appear to be sympatric (K. Wurdack, personal observation) and their local habitats differ in elevation, exposure and vegetation type. *Tryssophytonquadrifolius* would appear poorly adapted from an ecophysiology perspective as a summit endemic, with relatively large thin leaves, compared with much of the associated montane vegetation which has reduced sclerophyllous and/or coriaceous physiognomies as adaptations to cold and exposure (see Wurdack 2017). It is fundamentally terrestrial in nature with very similar rhizome and root morphologies to *T.merumense* and the observed epiphytism is likely opportunistic (i.e. a facultative epiphyte) in a very wet, dense-shrub habitat with sparse herbaceous understorey. The delicate leaf of *T.merumense* in transverse view shows dorsiventral differentiation and consists of a lamina up to six layers thick, organised as a single epidermal layer of thin-walled cells lacking thickened cuticles (adaxial epidermal layer of larger cells than the abaxial layer), a single layer of elongate tapered palisade parenchyma cells, rich in plastids and having considerable intercellular space towards their abaxial ends and a spongy parenchyma layer 2–3 cells thick (Fig. 4C, F). Stomata of the paracytic type are confined to the relatively smooth abaxial side (Fig. 4E) and large crystal druses occur occasionally in the mesophyll. The leaf ornamentation consists of short-stalked glandular trichomes (sensu classification of Wurdack [1986], but not surveyed there; Fig. 4A) and sparse adaxial scales (“sparsely strigulose,” according to Wurdack 1964, 1993), resembling erect horns that are typically collapsed in herbarium specimens (Fig. 4B, not collapsed due to CPD). These scales are 0.3–1 by 0.2 mm in size, loosely arranged in two files parallel to the midvein, multicellular, non-vascularised and have a rosette-like base of two undifferentiated cell layers (Figs 3J, 4B, D). While the scales and marginal teeth are superficially similar in form, the slightly shorter, fatter teeth terminate principal veins that traverse halfway into the tooth (i.e. submarginal principal vein termination; Figs 3J, 4B). The thin leaves of *T.quadrifolius* were not examined in transverse view, but clearly possess many of the same features including paracytic stomata and crystal druses; surface ornamentation is similar but the scales are slightly larger and only occur sparsely at the leaf tip. The teeth apices are also more prolonged in *T.quadrifolius* (0.5–0.9 mm long) than *T.merumense* (0.2–0.3 [0.4] mm). Species of *Boyania*, *Calvoa* and *Phainantha* variously have similar glandular trichomes along with long simple trichomes, but not the scales. In addition, other taxa formerly placed in the Bertolonieae (e.g. *Salpingaperuviana* [Cogn.] Wurdack and *Sarcopyramisnapalensis* Wall.) have glandular trichomes and/or scales.

Amongst reproductive features, the buds of both *Tryssophyton* species are red due to a pigmented layer of the outer (abaxial) exposed parts of the petals, which then open to reveal lighter inner surfaces. *Tryssophytonmerumense* has white inner (adaxial) petal surfaces and filaments, leaving the exterior strikingly bicoloured where the petals overlapped in bud (giving them a distinctive “candy-cane” pattern) and were still evident on the holotype (Fig. 3A–C). *Tryssophytonquadrifolius* differs slightly in floral colour with petals that are pink on the adaxial surfaces and pink filaments (Fig. 3D). The fruits and seeds of *Tryssophyton* have not been described due to mature fruit previously lacking (Wurdack 1964, 1993). When originally described, *T.merumense* (Wurdack 1964) was reported as 4-locular and mature fruits were not known. With additional collections made afterwards, here we can report that the fruits of *Tryssophytonmerumense* (*Wurdack 5870*, US) are of the angular capsule type and 3–4 locular. The mature capsule is ca. 3 mm tall by 3–5 mm wide on a thickened, tapering pedicel (totalling 15–18 mm long with capsule plus poorly differentiated pedicel), obscurely angular with twice as many ridges as locules (e.g. 6-sided if 3-locular), lacks a central column and crowned by stiff valves and short calyx lobes (Figs 2I, 3G, H). The valves are two per carpel (6 or 8 total per capsule), cartilaginous, 1.5 mm tall, triangular and attached at the capsule periphery such that the interior edges (septicidal and loculicidal splits) are free to flex for dispersal of the < 20 seeds loosely filling each locule. The fruit placenta remnants persist as basal-axillary clavate structures distally bearing fine fimbriae (Fig. 3G, H). Each fimbria is 0.2–0.4 mm long, terminated by a delicately attached seed and appears to consist of vasculature extending from the fruit placenta through a funiculus of uncertain length to the seed. At dehiscence, the erect fruit apex remains covered by the flexible valves.

Fruit and placenta morphology are remarkably diverse in Melastomataceae and phylogenetic evidence indicates complexity and homoplasy in fruit type evolution (Baumgratz 1983–1985, Clausing et al. 2000, Bacci et al. 2019). The broadly defined angular capsule fruit type, common in the Sonerileae-Dissochaeteae complex and many of its relatives, encompasses several distinct morphologies whose underlying developmental and structural differences are poorly understood. *Tryssophyton* presents its own fruit variant with the unusual features of basal-axile placentae, central column lacking and odd valves. Close relatives (i.e. *Calvoaorientalis* Taub., *J. Wurdack 2853*, US; *Phainanthalaxiflora* (Triana) Gleason, *Henkel 1689*, US; *Sarcopyramisnapalensis*, *Henry 13562c*, US) are mostly quite different, with axile placentae along a central column with seeds subsessile or on distinct short funiculi and fimbriae lacking. *Boyaniaayangannae* (*Henkel 147*, US) has more similarity with basal-axile fruit placentae, central column lacking and fimbriae present (funiculi to 0.1 mm, but fimbriae appearing longer due to frayed placental vasculature), although the overall fruit morphology differs in an apical glandular collar that lacks the stiff valves. In *Bertolonia*, the seeds are attached on long lateral placental branches of a usually distally-elaborated central column and have variable fimbriae that can be long (e.g. *B.acuminata* Gardner) or short to absent (e.g. *B.maculata* DC.) at dehiscence (see Baumgratz 1983–1985, 1989–1990). The long fimbriae – tips of a complex vascular skeleton – are also a combination of distally funicular and proximally placental origin and resemble those in *Tryssophyton*.

The seeds of *Tryssophytonmerumense* are 0.6–0.7 by 0.4–0.5 mm, ovoid, lack appendages and are minutely papillose with sinuous interdigitating testa cell patterning (Fig. 4G). The seeds of *T.quadrifolius* appear similar (see Description), but were immature in one partly dissected young fruit. They fall within the variable “bertolonoid” type and most closely resemble the seeds of *Triolena* Naudin (Whiffin and Tomb 1972, Bacci et al. 2019: fig. 7).

## Supplementary Material

XML Treatment for Tryssophyton quadrifolius

XML Treatment for Tryssophyton merumense

## Appendix 1

Sources for plastid data used in the phylogenetic analysis of *Tryssophyton*. Ordered as: Taxon, Voucher (new data only), GenBank numbers for *ndhF*, *rbcL* and *rpl16*, respectively. Dash (-) indicates missing data, asterisk (*) indicates taxa that presently appear under a synonym in GenBank and **bold** indicates new data.

Outgroups: *Alzateaverticillata* Ruiz & Pav., AF215591, -, AY151598. *Eugeniauniflora* L., AF215592, AF294255, AF215627. *Myrtuscommunis* L., AF215593, AF294254, AF215628. *Oliniaventosa* (L.) Cufod., AF215594, AF215546, -. *Penaeamucronata* L., AF270756, AJ605090, AF222782. *Rhynchocalyxlawsonioides* Oliv., AF270757, AF215547, AF215631. Melastomataceae: *Aciotispurpurascens* (Aubl.) Triana, AF215561, -, AF322231. *Allomaietaebejicosana* Lozano, JF831961, JF831986, JF832012. *Allomaietagrandiflora* Gleason, JF831962, JF831987, JF832013. *Allomaietahirsuta* (Gleason) Lozano, JF831963, JF831988, JF832014. *Allomaietapancurana* Lozano, JF831967, JF831993, JF832017. *Allomaietavillosa* (Gleason) Lozano, JF831969, JF831994, JF832019. *Allomaietazenufanasana* Lozano, JF831970, JF831995, JF832020. *Alloneuronulei* Pilg., JF831971, JF831996, JF832021. *Arthrostemmaciliatum* Pav. ex D.Don, AF215562, AF215522, AF215605. *Astroniasmilacifolia* Triana ex C.B.Clarke, AF215549, -, AF215596. “*Behuriacomosa* R.Tavares, Baumgratz & R.Goldenb.”, JQ899111, JQ899084, JQ899060. *Behuriaglutinosa* Cogn., JQ899112, JQ899085, JQ899061. *Belluciaaequiloba* Pilg., JF831972, JF831997, JF831997. *Belluciaarborescens* (Aubl.) Baill.*, EU711377, JQ626318, JF832035. *Belluciagrossularioides* (L.) Triana, EU711372, EU711385, JF832023. *Belluciamespiloides* (Miq.) J.F.Macbr.*, GU968822, KF781623, -. *Belluciapentamera* Naudin, AF215578, KF781624, AF215615. *Belluciaspruceana* (Benth. ex Triana) J.F.Macbr.*, GU968823, KF781625, -. *Bertoloniamargaritacea* Naudin*, JQ899130, AF215512, JQ899080. *Bertoloniamosenii* Cogn., JF831973, JF831998, JF832024. *Blakeagracilis* Hemsl., JF831974, JF831999, JF832025. *Blakeamultiflora* D.Don*, JQ899132, JQ899107, JQ899082. *Blakeaschlimii* (Naudin) Triana, EU711373, EU711386, JF832026. *Blakeatrinervia* L., AF215555, AF215516, AF215600. *Blakeawatsonii* (Cogn.) Penneys & Almeda, JQ899133, JQ899108, JQ899083. *Blastusborneensis* Cogn. ex Boerl., AF215585, -, AF215621. *Blastuscochinchinensis* Lour., KX066244, KP094575, KM521849. *Blastuspauciflorus* (Benth.) Guillaumin, KX066245, KP095022, KM521850. *Boyaniacolombiana* Humberto Mend., -, JQ899086, JQ899062. *Brasilianthuscarajensis* Almeda & Michelang., KX765168, KX765169, KX765170. *Brediafordii* (Hance) Diels, KT354883, KT354892, KM521851. *Brediasessilifolia* H.L.Li, -, KP094838, -. *Calvoagrandifolia* Cogn., -, AY667151, AY660632. *Cambessedesiaeichleri* Cogn., JQ899113, JQ899087, JQ899063. *Cambessedesiaespora* (A.St.Hil. ex Bonpl.) DC., JQ899114, JQ899088, JQ899064. *Cambessedesiahilariana* (A.St.Hil. ex Bonpl.) DC., JQ899115, JQ899089, JQ899065. *Cambessedesiamembranacea* Gardner, AY553782, -, AY553775. *Castratellapiloselloides* (Bonpl.) Naudin, AY553783, AY553779, AY553774. *Centradeniainaequilateralis* (Schltdl. & Cham.) G.Don, AF215563, EU711387, AF215606. *Chalybeamacrocarpa* (Uribe) Penneys & Morales-P, JQ899121, JQ899095, JQ899071. *Comoliamicrophylla* Benth., JF831975, JF832000, JF832028. *Desmoscelisvillosa* (Aubl.) Naudin, EU711374, EU711389, JF832029. *Dichaetantheraarborea* Baker, AF272800, -, AF294470. *Dichaetantheraasperrima* Cogn., AF215564, AF215523, AF215607. *Diplectriadivaricata* (Willd.) O.Ktze, AF215556, AF270746, AF215601. *Dissochaetabracteata* (Jack) Blume, AF289369, -, AF294471. *Dolichouraspiritusanctensis* Brade, JQ899116, JQ899090, JQ899066. *Driesseniaglanduligera* Stapf, AF215586, AF270749, AF215622. *Eriocnemafulva* Naudin, AY553781, AY553777, AY553772. *Fordiophytonbrevicaule* C.Chen, KT354884, KT354893, KM521852. *Fordiophytonchenii* S.Jin Zeng & X.Y.Zhuang, KT354886, KT354895, KM521854. *Fordiophytoncordifolium* C.Y.Wu ex C.Chen, KT354885, KT354894, KM521853. *Fordiophytonfaberi* Stapf, KU208089, KU208090, KM521855. *Fordiophytonhuizhouense* S.Jin Zeng & X.Y.Zhuang, KT354887, KT354896, KM521856. *Fordiophytonpeperomiifolium* (Oliv.) C.Hansen, KT354888, KT354897, KM521857. *Fordiophytonzhuangiae* S.Jin Zeng & G.D.Tang, KX066246, -, KX037425. *Graffenriedalatifolia* (Naudin) Triana, AM235411, AM235644, AM235447. *Graffenriedamoritziana* Triana, EU055944, EU711390, JF832031. *Graffenriedarotundifolia* (Bonpl.) DC., AF215576, AF215532, AF215613. *Henrietteamartiusii* (DC.) Naudin, EU711375, EU711391, JF832032. *Henrietteapatrisiana* DC., JF831977, JF832002, JF832033. *Henriettearamiflora* (Sw.) DC., GU968811, KF781627, -. *Henrietteasuccosa* (Aubl.) DC., GU968815, KF781628, -. *Henrietteatuberculosa* (Donn. Sm.) L.O.Williams*, GU968816, KF781629, -. *Heterocentronelegans* (Schltdl.) Kuntze, AF272804, AY456135, AF325926*Heterocentronsubtriplinervium* (Link & Otto) A.Braun & C.D.Bouché, AF215566, AF270747, AF210374. *Heterotisfruticosa* (Brenan) Veranso-Libalah & G.Kadereit, AF272802, -, AF210377. *Heterotisrotundifolia* (Sm.) Jacq.-Fél., AF215565, U26323, AF270745. *Huberiaconsimilis* Baumgratz, JQ899117, JQ899091, JQ899067. *Huberiaperuviana* Cogn., JQ899118, JQ899092, JQ899068. *Lavoisieracordata* Cogn., AF215582, AF215540, AF210371. *Lavoisierapulchella* Cham., EU711376, EU711392, JF832034. *Macairearadula* (Bonpl.) DC., EU711378, EU711394, JF832036. *Macrocentrumcristatum* (DC.) Triana, AM235412, AM235645, AM235448. *Macrocentrumrepens* (Gleason) Wurdack, AF215551, AF215513, AF215598. *Medinillaalternifolia* Blume, AF289374, -, AF322229. *Medinillahumbertiana* H. Perrier, AF215557, AF215517, AF215602. *Melastomabeccarianum* Cogn., AF272805, AM235646, AM235449. *Melastomacandidum* D.Don, AB436365, GQ436728, AF215608. *Melastomadodecandrum* Lour., AF272808, GQ436727, -. *Melastomamalabathricum* L., AF272810, AF270748, AB436376. *Melastomasanguineum* Sims, AF270754, HQ415218, AB436378. *Melastomatetramerum* Hayata, AB436364, -, AB436373. *Memecylondurum* Cogn., AM235408, AM235641, AM235444. *Memecylonedule* Roxb., AF215574, AF215528, AF215609. *Merianiamacrophylla* (Benth.) Triana, AM235414, AM235647, AM235450. *Merianianobilis* Triana, AF215577, AF215533, AF215614. *Merianiaphlomoides* (Triana) Almeda, EU055971, EU711395, JF832037. “*Meriantherabullata* R.Goldenb., Fraga & A.P.Fontana”, JQ899129, JQ899104, JQ899078. *Meriantheraburlemarxii* Wurdack, JQ899122, JQ899096, JQ899072. “*Meriantheraparvifolia* R.Goldenb., Fraga & A.P.Fontana”, JQ899127, JQ899102, JQ899077. *Meriantherapulchra* Kuhlm., JQ899124, JQ899098, JQ899073. *Meriantherasipolisii* (Glaz. & Cogn.) Wurdack, JQ899126, JQ899100, JQ899075. “*Meriantheraverrucosa* R.Goldenb., Fraga & A.P.Fontana”, JQ899125, JQ899099, JQ899074. *Miconiabicolor* (Mill.) Triana*, EU056130, KX397981, -. *Miconiacalycina* Cogn., EU056001, JF832003, JF832038. *Miconiacubatanensis* Hoehne, EU056020, -, -. *Miconiadodecandra* (Desr.) Cogn., EU056026, EU711396, JF832039. *Miconiadonaeana* Naudin, AM235415, AM235648, AM235451. *Miconiafasciculata* Gardner, EU056033, -, -. *Miconiamayeta* (D. Don.) Michelang.*, AF215581, AF215537, AF215618. *Miconiapetiolaris* (Schltdl. & Cham.) Michelang., AM235410, AM235643, AM235446. *Miconiarubra* (Aubl.) Mabb.*, AF215579, AF215535, AF215616. *Miconiasecunmexicana* G.Ocampo & Almeda*, AF215580, AF215536, AF215617. *Miconiatococa* (Desr.) Michelang.*, AM235417, AM235650, -. *Miconiaurbani* (Cogn.) Cogn.*, AF270753, AF215538, AF215619. *Monochaetumcalcaratum* (DC.) Triana, AF215568, AF215524, AF210372. *Monolenaprimuliflora* Hook. f., AF215552, AF215514, AF270743. *Mouriricrassifolia* Sagot, -, FJ038111, -. *Mouririguianensis* Aubl., AF215575, AF215529, AF215610. *Mouririhelleri* Britton, AF322230, AF270752, AF215611. *Nepseraaquatica* (Aubl.) Naudin, AF215569, JQ592692, AF210373. *Osbeckiachinensis* L., AF215570, AF215525, AF210378. *Osbeckiastellata* Buch.-Ham. ex Ker Gawl., AF272818, U26330, -. *Oxysporapaniculata* (D.Don) DC.*,, KX527089, MH722293. *Phainanthalaxiflora* (Triana) Gleason, JF831980, JF832006, JF832043. *Phainanthashuariorum* C.Ulloa & D.A.Neill, JF831981, JF832007, JF832044. *Phyllagathisgymnantha* Korth., AF215590, -, AF215626. *Phyllagathishispidissima* (C.Chen) C.Chen, KT354889, KT354899, KM521858. *Physeterostemonfiaschii* R.Goldenb. & Amorim, EU711379, EU711397, JF832045. *Physeterostemonjardimii* R.Goldenb. & Amorim, EU711381, EU711399, JF832046. “*Physeterostemonthomasii* Amorim, Michelangeli & R.Goldenb.”, EU711383, EU711401, JF832047. *Pternandracoerulescens* Jack, AF215558, AF215518, AF322232. *Pternandraechinata* Jack, AF215559, AF215520, AF270744. *Pternandramultiflora* Cogn., AF215560, -, AF215603. *Pterolepisglomerata* (Rottb.) Miq., AF215571, AF215526, AF210376. *Rhexiamariana* L., AF272819, KJ773817, AF323723. *Rhexiavirginica* L., AF215587, MG248427, AF215623. *Rhynchantheragrandiflora* (Aubl.) DC., AF215584, AF215542, AF210369. “*Rupestreajohnwurdackiana* (Baumgratz & D’El Rei Souza) Michelang., Almeda, & R.Goldenb.”, KM373899, KM373900, KM373901. *Salpingamaranonensis* Wurdack, JF831982, JF832008, JF832048. *Salpingasecunda* Schrank & Mart. ex DC., EU711384, EU711402, JF832049. *Sarcopyramisparvifolia* Merr. ex H.L.Li, -, KX527237, -. *Siphantherapaludosa* (DC.) Cogn., -, AY553780, AY553776. *Tibouchinagrossa* (L.f.) Cogn., JF831983, JF832009, JF832050. *Tibouchinalongifolia* (Vahl) Baill., AF215572, JQ592704, AF210375. *Tibouchinaurvilleana* (DC.) Cogn., AF272820, U26339, AF322234. *Tigridiopalmamagnifica* C.Chen, KT354891, KT354900, KM521859. *Triolenaamazonica* (Pilg.) Wurdack, JF831984, JF832010, JF832051. *Triolenapaleacea* (Triana) Almeda & Alvear, JF831976, JF832001, JF832030. *Triolenapustulata* Triana*, JQ899135, JQ899110, AF215599. *Tristemmalittorale* Benth., -, AY667150, AY660631. *Tristemmamauritianum* J.F.Gmel., AF272821, -, AF322233. *Tryssophytonmerumense* Wurdack, *A. Radosavljevic 165* (US), **MK284234**, **MK284232**, -. *Tryssophytonquadrifolius* K.Wurdack & Michelang., *K. Wurdack 5865* (US), -, **MK284233**, -. *Warneckeamembranifolia* (Hook. f.) Jacq.-Fél., AF331711, KC628335, -. *Wurdastomcuatrecasasii* (Wurdack) B.Walln., JF831985, JF832011, -.
